# A biogeographical study of red listed lichen species at temporal and spatial scales within protected and non-protected areas

**DOI:** 10.1038/s41598-022-04872-1

**Published:** 2022-01-18

**Authors:** Ioana Vicol, Simona Mihăilescu

**Affiliations:** Department of Ecology, Taxonomy and Nature Conservation, Institute of Biology Bucharest of Romanian Academy, 296 Splaiul Independentei, P.O. Box 56–53, 060031 Bucharest, Romania

**Keywords:** Plant sciences, Environmental sciences

## Abstract

The present study is focused on the temporal and spatial distribution of red listed lichen (RLL) species identified in both non-protected areas (NPAs) and protected areas (PAs) in Romania. This study revealed different scenarios of RLL based on two major patterns: (1) the fate of RLL before and after their designation in the red list in Romania and (2) the fate of RLL before and after the designation of PAs in Romania. Generally, the occurrences of RLL were well represented in time and space in both NPAs and PAs through geomorphological and biogeographical units. In particular, the occurrences of RLL were well represented, especially in hilly areas within PAs before and after their official designation, and this was an important aspect over a long period of time. Although NPAs were not less important regarding the occurrences of RLL species, it was observed that they followed the same pattern as PAs, namely, they were well represented in hilly areas and over a long period of time. The bioregions were significant for RLL species in both NPAs (continental and stepic) and PAs (continental, panonic, and stepic). As a significant finding of this study, NPAs and PAs should be subjected to an adequate conservation regime due to their biotical traditional heritage.

## Introduction

Protected areas are important for worldwide biodiversity conservation and they involve serious efforts to conserve habitats and their associated species^1–3^. In contrast, non-protected areas (NPAs) are subjected to a major impact of anthropogenic activities, and researchers do not pay much attention to them^1,2,4–6^. Socioeconomic development has destroyed important areas for biodiversity^7^ and will continue to affect both biodiversity and habitats on a large spatial scale^8–10^. Species diversity could be higher in NPAs than in protected areas (PAs); moreover, NPAs also have value for biodiversity, and therefore, these areas should not simply be allowed to disappear due to environmental anthropogenic changes^2,4,5,8^. Maintaining high-quality habitats within both NPAs and PAs is important for the survival of RLL species^1,10^. Conservation of RLL species could be realized by creating a balance of conservation actions and economic gains in both NPAs and PAs^10^. Another important aspect is represented by forest productivity capacity related to natural resources, which could represent a core for biodiversity and therefore could be important for habitat conservation^11^.

Efforts to conserve native conditions of natural habitats that enhance the survival of RLL species are problematic^10^. However, the difficult task of securing biodiversity could be achieved by applying already available efficient management measures^12–14^. The conservation of RLL species requires microhabitat and macrohabitat diversity in both NPAs and PAs^6,9,10^.

Despite the fact that biodiversity is threatened at all organizational levels by numerous human activities, over time, greater attention has been given to animals (especially birds and mammals) and vascular plants. Cryptogams such as lichen have been ignored in most conservation programs^4,5^.

Throughout time, Romanian natural habitats have been subjected to great fragmentation, restricting biodiversity to small areas often lacking connectivity^15,16^. Furthermore, the change in the ownership of NPAs and PAs in Romania, especially after the fall of the communist regime, has caused environmental modifications that have affected their resources^17^. Europe contains forests characterized by a native and old structure with an insignificant influence by anthropogenic activities^18,19^. In Romania, one native and multiaged forest area with an absolute protection regime is the Slătioara Natural Reserve, characterized by the high diversity of the structure and composition of its vegetation^20^.

The aim of this study was to assess the RLL occurrences within geomorphological units and biogeographical regions over time within and between the PAs and NPAs of Romania. In particular, we analysed (1) the RLL occurrences before and after designation of the PAs across geomorphological and biogeographical attributes; (2) the RLL occurrences before and after their designation in the Romanian RLL, taking into account their geomorphological and biogeographical attributes; and (3) whether the pattern of RLL occurrences during the time periods and geographical attributes were more significant for PAs than NPAs.

## Results

The RLL species were identified in 92 sites within PAs and in 99 sites within NPAs. Among a total of 18 RLL species, all were found in the PAs, while in the NPAs, 15 RLL species were identified (supplementary file: Table S1 and Table S2). The distribution and number of RLL species within the geomorphological units and biogeographical regions in both NPAs and PAs are given in supplementary file: Table S3. The occurrence of each RLL species in the geomorphological unit and biogeographical region in the four periods of time are presented within the NPAs (supplementary file: Table S4) and PAs (supplementary file: Table S5).

Regarding the statistical analysis, it was important to control confounders based on one-way NPMANOVA for RLL species data within NPAs and PAs. After removal, the data belonging to confounders such as *Bryoria lanestris* (Ach.) Brodo & D. Hawksw. and *Nephromopsis chlorophylla* (Willd.) Divakar, A. Crespo & Lumbsch, the results of this test based on the adjustment of confounders did not indicate significant associations between datasets represented by the other RLL species within the NPAs and PAs (F = 3.76; *p* = 0.06). This indicates that the results from the following statistical analyses were not biased by associations between the datasets.

In the present study, the RLL species number did not significantly differ among the NPAs and PAs (chi^2^ = 2.75; p = 0.99).

Within the NPAs, the time periods 1850–1900 and 2001–2020 were excluded from statistical analyses due to spatial autocorrelation results, because of these time periods and their associated geomorphological and biogeographical units were biased by associations between the datasets. Also, within the PAs, the time periods 1850–1900 and 1901–1950 were excluded from statistical analyses based on the same statement as above.

In the seriation analysis, the RLL species were ordered across geomorphological and biogeographical units throughout the time periods. Thus, throughout the time periods, the ordered pattern of RLL species across the geomorphological and biogeographical units was significant within both the NPAs and PAs (Table 1). Within the NPAs, the RLL species were ordered in the hilly areas within continental (CON) during the second period of time and within stepic (STE) and CON bioregions during the third period of time (Table 1). Within the PAs, the RLL species were ordered in the third and fourth periods of time in the hilly areas situated in the CON and panonic (PAN) bioregions (in the third periods of time) and in the STE and CON bioregions in the fourth period of time (Table 1).

**Table 1:** The occurrence of RLL species within geomorphological units and biogeographical regions ordered over time periods (according to seriation analysis performed in PAST software).

The status of the studied areas	The occurrences of RLL species in geomorphological units and biogeographical regions during time				Statistics (Monte Carlo simulation)		
	1850–1900	1901–1950	1951–2000	2001–2020	M ± SD	Z score	*p* value
Non-protected areas	–	Hill, CON	Hill, CON, STE	–	0.53 ± 0.04	2.33	0.01
Protected areas	–	–	Hill, CON, PAN	Hill, STE, CON	0.52 ± 0.04	2.43	0.01

CON, continental bioregion; PAN, panonic bioregion; STE, stepic bioregion; –, a lack of data.

The occurrences of RLL species changed along spatial and time attributes. Thus, in the NPAs, major changes over time periods (1901–1950 and 1951–2000) in the occurrences of RLL species were found in geomorphological units such as hills and their corresponding CON and STE biogeographical regions (Table 2). In the NPAs, the RLL species were concentrated mostly in the CON bioregion during the second half of the twentieth century (Table 2). In the PAs, main changes over time periods (1951–2000 and 2001–2020) in the occurrences of RLL species were found in geomorphological units, such as hills and their corresponding CON, PAN and STE biogeographical regions (Table 3).

**Table 2:** The ordering of RLL species in the geomorphological units and biogeographical regions over time within NPAs (according to the seriation analysis performed in PAST, based on the unconstrained algorithm).

Species	STE	3	Hill	CON	2
*Ramalina obtusata* (Arnold) Bitter	*	*	*	*	
*Cetraria islandica* subsp. *islandica* (L.) Ach		*	*	*	
*Dolichousnea longissima* (Ach.) Articus		*	*	*	
*Melanelixia subaurifera* (Nyl.) O. Blanco, A. Crespo, Divakar, Essl., D. Hawksw. & Lumbsch		*	*	*	
*Peltigera lepidophora* (Vain.) Bitter		*	*	*	
*Lobaria pulmonaria* (L.) Hoffm.		*	*	*	*
*Stereocaulon alpinum* Laurer		*	*	*	*
*Usnea fulvoreagens* (Räsänen) Räsänen			*	*	*

2–1901–1950; 3–1951–2000; asterisks indicate the occurrence of RLL species in the geomorphological units and biogeographical regions over time; empty cells indicate no available data.

**Table 3:** The ordering of RLL species in the geomorphological units and biogeographical regions over time within the PAs (according to the seriation analysis performed with the PAST software, based on unconstrained algorithm).

Species	4	STE	HILL	CON	3	PAN
*Hypotrachyna sinuosa* (Sm.) Hale	*	*	*	*		
*Ramalina obtusata* (Arnold) Bitter		*	*	*	*	
*Cladonia macrophylla* (Schaer.) Stenh			*	*	*	
*Cladonia incrassata* Flörke			*	*	*	
*Dolichousnea longissima* (Ach.) Articus			*	*	*	
*Lathagrium dichotomum* (With.) Otálora, P.M. Jørg. & Wedin			*	*	*	
*Cetraria islandica* subsp. *islandica* (L.) Ach			*	*	*	
*Lobaria pulmonaria* (L.) Hoffm			*	*	*	*

3–1951–2000; 4–2001–2020; asterisks indicate the occurrence of RLL species in the geomorphological units and biogeographical regions over time; empty cells indicate no available data.

*The Kruskal–Wallis test* revealed significant differences between occurrences of RLL species for NPAs within geomorphological units and biogeographical regions over time periods (chi^2^ = 20.64; *p* = 0.0001) and for geomorphological units and biogeographical regions within PAs (chi^2^ = 15.55; *p* = 0.001).

*Post hoc comparison tests* pointed out significant differences in RLL species occurrences across biogeographical regions during the second and the third periods of time (STE versus CON, *p* = 0.0008) for NPAs. Within hilly areas from NPAs, significant differences in RLL species identified in the STE bioregion were recorded during the second and the third periods of time (hill versus STE, *p* = 0.0007).

*Post hoc comparison tests* performed for PAs indicated significant differences between pairwise data represented by RLL species across both geomorphological units and biogeographical regions during the third and the fourth periods of time. Furthermore, in the PAs, significant differences were obtained based on post hoc Mann–Whitney pairwise comparisons of RLL species occurrences within biogeographical regions during the third and the fourth periods of time (STE versus CON *p* = 0.003; CON versus PAN *p* = 0.0008). During the third and the fourth periods of time within hilly areas from PAs were recorded significant differences in RLL species identified in the STE and PAN bioregions (hill versus STE, *p* = 0.003; hill versus PAN, *p* = 0.0007).

In the NPAs, the occurrences of the RLL species within the geomorphological units and biogeographical regions were significantly different over the time periods but not in the periods of time when were designated in the Red List of lichens in Romania (supplementary file: Table S6). Otherwise, regarding the impact of the designation of PAs on RLL species, it was demonstrated that in PAs, the RLL species showed significant differences during the third (1951–2000) and fourth (2001–2020) periods of time within the geomorphological units and the biogeographical regions. Consequently, the RLL species showed significant differences in their occurrences before and after legal designation of the PAs (between 2000 and 2010) in Romania. In the time period 2007–2013, the RLL species for Romania were published and designated (supplementary file: Table S6). Based on the information presented in supplementary file: Table S6, 18 RLL species were designated in Romania^21,22^. In the PAs, the RLL species were significantly differentiated within geographical attributes before and after their designation in the two Red Lists (Tables 1, 3), especially before and after the designation of PAs (the PAs were designated in 2000 enforced by National Law no. 5/2000^23^, and 2007–2010 enforced by 2000 Natura network legislative framework^24,25^.

**Figure 1:**
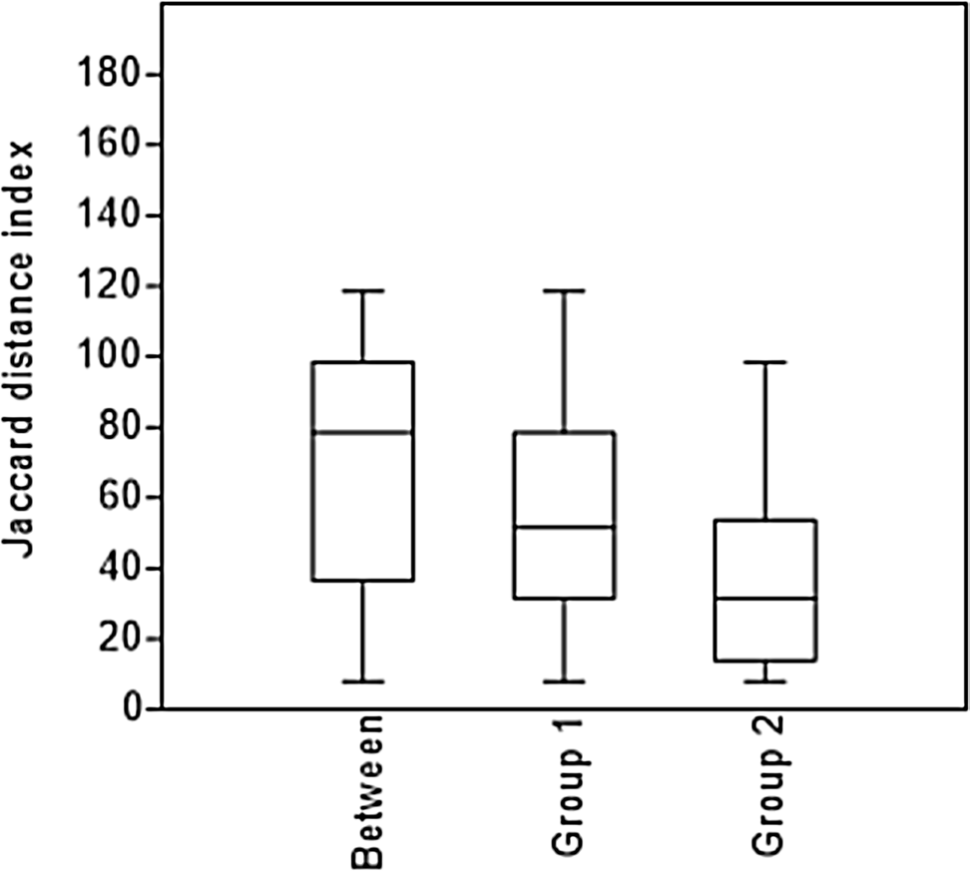
One-way ANOSIM box plot based on the two datasets represented by the occurrences of RLL species between NPAs and PAs (Group 1 is represented by PAs, and Group 2 is represented by NPAs).

*One-way ANOSIM* indicated significant differences in the RLL species between the NPAs and PAs (R = 0.41; *p* = 0.006) based on their occurrences in the geomorphological units and biogeographical regions over the various time periods (Fig. 1).

The post hoc pairwise ANOSIM test indicated significant differences (*p* = 0.006) in the occurrences of RLL species between all pairs of the two studied groups defined by the NPAs and PAs in their geomorphological units and biogeographical regions over the time periods.

The statistical analysis based on the Kolmogorov–Smirnov test indicated nonsignificant differential collection rates during all periods of time and their associated biogeographical and geomorphological variables within the NPAs and PAs. The one-way NPMANOVA did not indicate significant differential collection rates between the NPAs and PAs during all periods of time and their associated biogeographical and geomorphological variables.

## Discussion

In the present study the species number among NPAs and PAs was not significantly different. However, other studies showed that the species number was better represented in PAs than in NPAs, due to effective control of human activities^5^. Nevertheless, NPAs have particular importance for biodiversity conservation and support measures for sustainable management and limitations of anthropogenic pressure should be implemented^2,4,14^. Usually, red lists include threatened and extinct species with great importance for environmental resource conservation^4,26,27^. Red-listed lichen species are related to ancient habitats that contain specific microhabitats^28,29^ and therefore it is necessary to secure RLL survival by adequately protecting these habitats^10^. The occurrence of RLL species across different biogeographical regions both in NPAs and PAs indicate that these are widely adapted to different environmental conditions offered by a wide variety of habitats which are characterized by different geographical and climatic aspects^34^.

At small spatial scales, geomorphological units such as hilly areas significantly affect the variation in the occurrences of RLL species; therefore, geomorphology associated with different climatic conditions (mountain habitats with low temperature and high amounts of precipitation are different from hilly and plain habitats with high temperatures and low amounts of precipitation) is an important driver of various tree species^30^ that harbour RLL species due to tree diversity and microhabitat quality^3,10^. At a large spatial scale, geomorphology and biogeographical regions and their associated abiotic parameters play an important role in determining the occurrence of lichen species across various habitats^13^. It is worth considering that even at higher altitudes in mountain areas, the anthropogenic pressure related to a weak efficacy of legislative systems has a negative impact on natural habitats^17^, which leads to many difficulties in biodiversity conservation^31^. PAs should be represented by a wide range of geomorphological features and the sustainable connectivity needed for the long-term conservation of biodiversity^32^.

The observed distribution of RLL species occurrences across landscape features, such as the structural heterogeneity of traditional habitats in NPAs and PAs, is attributed to the lack of anthropogenic impact^33^. Within these remote areas, the main management land use type is based on traditional practices that do not represent a major negative impact on the environment^33^. In Romania, many NPAs are situated in remote sites with long-term continuity and connectivity of different types of habitats, such as old-growth forests, meadows, rocks, wetlands, etc.^34^. The structural complexity of the traditional landscape represents suitable habitats for lichen species and therefore should be a focus for the establishment of PAs in Romania^35^. Habitat connectivity among protected landscapes and their heterogeneity are important criteria, especially for threatened species and their habitats^36,37^.

Within this study, the RLL species were significantly represented during various periods of time across NPAs and PAs. As in this study, both NPAs and PAs are important for RLL species^6^, which emphasizes their importance in biodiversity conservation^38^. The different geographical attributes (geomorphology and biogeographical regions) that characterize PAs support a high richness of lichen species^8^. In this regard, the territory of Romania is well represented by geomorphological units^30^ and biogeographical regions^34^, and RLL species have been identified in various vegetation communities^22,39–43^.

In many regions of the world, ancient habitats are restricted and isolated areas^8,44^ surrounded by a matrix of strongly disturbed anthropogenic areas^27,38^, which significantly affects lichen communities^43,45^. The connectivity of habitats is an important attribute for biodiversity conservation within PAs^44^ during dynamic landscape stages and it impacts species assemblages and the quality of their habitats^46^.

The RLL species represent a natural heritage because some of them, such as *Lobaria pulmonaria* (L.) Hoffm., are widely recognized as valuable indicators of ancient forests threatened by industrial activities and the management of forested areas^47^. Furthermore, *L*. *pulmonaria* is an RLL species in many countries in Europe^47,48^, and it is listed as endangered in Europe^49^.

The support of biodiversity conservation is highlighted by a complex frame of concepts and strategies based on historical arguments with their origins in scientific and political resolutions^50^. It is important to grasp that the designation of PAs does not represent a universal remedy for biodiversity conservation; consequently, biodiversity conservation within PAs depends on sufficient ecological resources secured by low human pressure^27^. In European countries, the conservation of biodiversity in the current context could not completely eliminate anthropogenic impacts, but it could be reduced so that the species and habitats are not affected or lost^51^. The common efforts of politicians and local communities to advance strategies for long-term environmental sustainability are key to the success of nature conservation efforts^50,51^.

### Conclusions

Non-protected areas are important for RLL species; therefore, these areas should be subjected to adequate management for the long-term conservation of habitats and their associated RLL species. Additionally, PAs and their geographical attributes have an important role for RLL species. Through time, the occurrence of RLL species has been significantly changed, and particular geographical patterns have emerged that support the habitat of these important species in NPAs and PAs. The habitats represented by NPAs and PAs are both equally important for conservation of the RLL species in Romania. The time periods clearly suggest that it does not matter when the PAs were designated but how well their traditional natural heritage was conserved. This statement is supported by the fact that the RLL species survived over time, and more importantly, these species are still found within NPAs and PAs in Romania.

## Methods

### Legislative framework

The RLL species from Romania are not subjected to any legislative framework. The designation of PAs is mainly enforced by Law no. 5/2000^23^ as a tool of national legislative support for habitats and species conservation. Additionally, the other PAs were designed by the Natura 2000 Network^24,25^.

### Data sources

Data on the time and spatial occurrences of RLL species were obtained from the Mycological Herbarium of the Institute of Biology from Bucharest (BUCM), which belongs to the Romanian Academy (supplementary file: Table S1 and Table S2), and the author’s unpublished and published data (supplementary file: Table S1 and Table S2). For this study, all RLL species from Romania were considered, for a total of 18 species (supplementary file: Table S6). The nomenclature of the lichen species is according to www.indexfungorum.org.

### Assessment of time periods

In this study, four different time periods were considered: the second half of the XIX century (1851–1900), the first and the second half of the XX century (1900–1950 and 1951–2000, respectively), and the first decades of the first half of the XXI century (2001–2020). Each period of time is equal to 50 years with the exception of the first decades of the first half of the XXI century.

### Assessment of spatial areas

Spatial areas were represented by three major geomorphological units: mountain, hill, plain and all available biogeographical regions: alpine (ALP), continental (CON), panonic (PAN), pontic (PON), and stepic (STE). Within this study, Marine Black Sea (MBLS) and Black Sea (BLS) biogeographical regions did not include available datasets. Small spatial scales are represented by each geomorphological unit (plain, hill, mountain) and each biogeographical region (alpine, continental, panonic, pontic, stepic), while large spatial scales are represented by all geomorphological units and biogeographical regions across Romanian territory.

### Geographical distribution of RLL species in Romania

The RLL species were georeferenced based on a geographical information system represented by geographical coordinates measured in decimal degrees within the studied NPAs and PAs, which were widely spread across a range of different geomorfological units, such as plains, hills, and mountains.

The old chronological data included in supplementary file: Table S1 and Table S2 of RLL species were represented by toponyms and localities (towns and villages). The geographical distribution of RLL species used in this study in the case of old chronological data, which includes toponyms and localities, was based on the geographical information system represented by the Universal Transverse Mercator (UTM) grid system of 100 × 100 km for Romania^52,53^. A universal transverse mercator grid was used to identify toponyms and localities in Romania in the case of old chronological data; most toponyms and localities were published by^52,53^ based on a UTM grid. The recent chorological data included in supplementary file: S1 and Table S2 taken into account in this study were also represented by toponyms and localities and were geographically referenced using GPS coordinates based on degrees, minutes, and seconds. An exception is the newer records that were determined using a GPS set to geographical coordinates measured in decimal degree to give a more precise location for plotting. The spatial resolution of some older data represented by toponyms is 100 km square whilst the spatial resolution of newer data is represented by a few meters due to the accuracy of handheld GPS. In the database of this study, the entire dataset represented by old and recent chronological data was converted from UTM geographical coordinates and geographical coordinates given as degrees, minutes, and seconds in the decimal degree World Geodetic System of 1984 (WGS84) using the option Military Grid Reference System (MGRS)/UTM reference (UTMREF) based on WGS84 available at the following site https://coordinates-converter.com.

**Figure 2:**
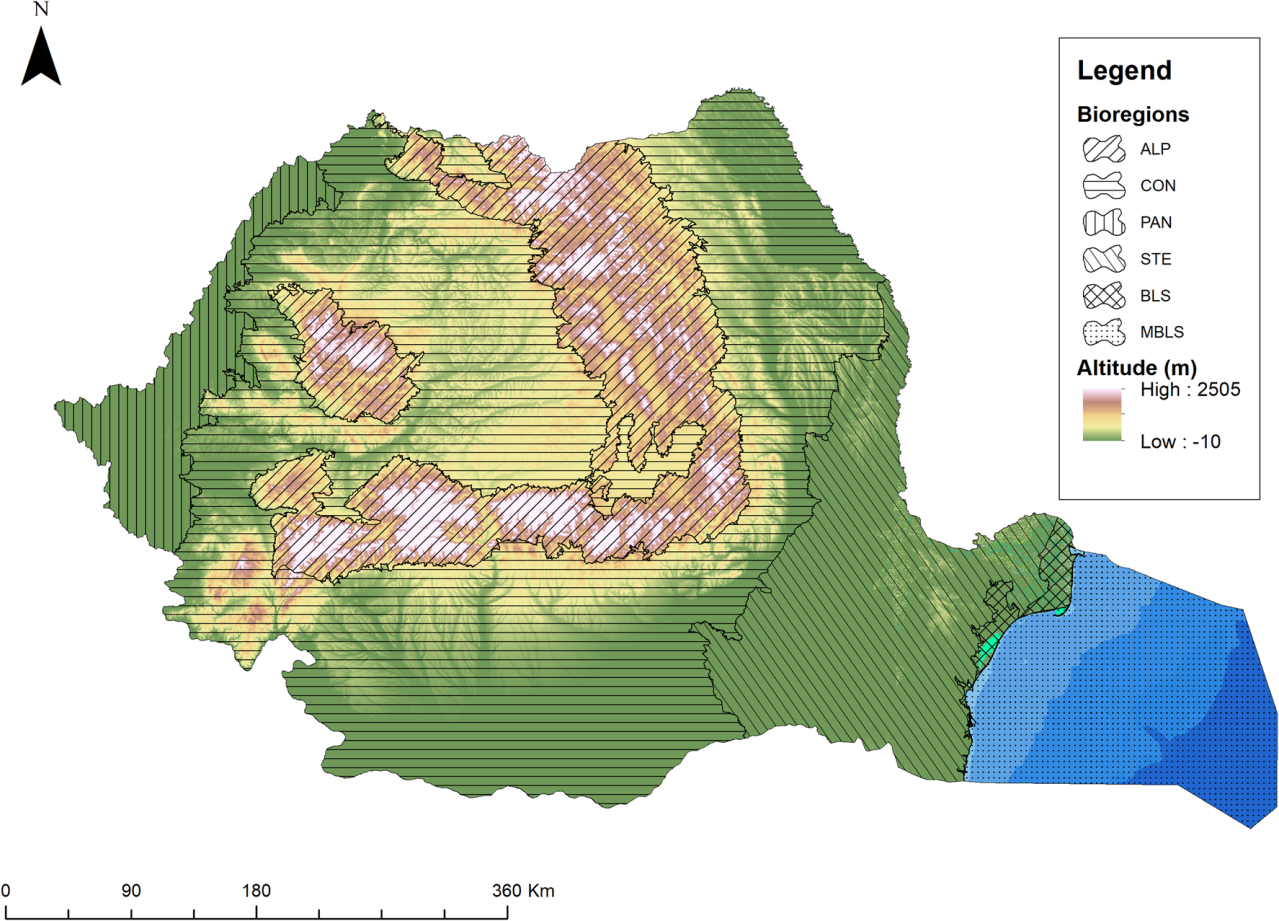
The geomorphological units and biogeographical regions of Romania (the map was generated using ArcGIS software, version 10.4, provided by Environmental Systems Research Institute, Redlands CA, 2013^54^).

A map that shows the geomorphological units and biogeographical regions from Romania (Fig. 2) was generated based on the decimal degree (WGS84) geographical coordinates in ArcGIS 10.4^54^ using the ETRS89 LAEA reference system. Elevation is represented based on the 30 m digital elevation model over Europe freely available from the Copernicus Land Monitoring Service website (https://land.copernicus.eu). Biogeographical regions were drawn using the shapefile available on the website of the Romanian Ministry of Environment, Water and Forestry (http://www.mmediu.ro) using the colour codes specified by the European Environmental Agency on their website (https://www.eea.europa.eu).

Within this work, in the case of old records (published data, especially in the last two centuries), it is not known how the data were collected by authors to avoid spatial biases such as data collected near roads due to easy accessibility. Based on this idea, in the author’s original database, most data on the geographical distribution of RLL species in Romania were collected in remote areas (far from roads and localities) to avoid spatial accessibility biases. There were some exceptions in the case of one specimen of *Hypotrachyna sinuosa* (Sm.) Hale, which was collected near a national road in the southern part of Romania (all of the other specimens of *H*. *sinuosa* considered in this study were collected in remote areas). Additionally, another exception was the case of three specimens of *Cetraria islandica* subsp. *islandica* (L.) Ach. collected near a national road in the central part of Romania. In regards to the old records, because it is not known how the data were collected, to avoid spatial biases (near roads and certain localities) a statistical analysis in this sense was impossible.

### Statistical analysis

Lichen occurrence assessment was established as the presence/absence of a species recorded within geomorphological units and biogeographical regions through time periods within PAs and NPAs.

In the statistical analysis, an adjustment was performed to control all of the confounders so that the results of the statistical analyses reflected true statements^55^. In this regard, a one-way NPMANOVA test was performed to control for the confounders^55^. In the one-way NPMANOVA test, the distance measure was based on the Kulczynski index appropriate for binary data. The test was computed by 9999 permutations, and finally, a post hoc test was provided between the pairwise of two groups represented by PAs and NPAs^56^. The one-way NPMANOVA test identified two confounders based on the data of two RLL species, *Bryoria lanestris* (Ach.) Brodo & D. Hawksw. and *Nephromopsis chlorophylla* (Willd.) Divakar, A. Crespo & Lumbsch, and as a consequence, the data for these species were eliminated from all statistical analyses.

*Spatial autocorrelation* was used to identify the dependence among the spatial data (geographical coordinates of the RLL species) and the biogeographical and geomorphological data of the RLL species within both NPAs and PAs^57^. Because the dataset is represented by binary data (presence/absence of RLL species), it is recommended to analyse spatial autocorrelation by using generalized estimating equations^58^. The generalized estimating equations were performed with R software^59^ by using the geepack package^60–62^. The generalized estimating equations indicated a significant correlation within the spatial data represented by geographical coordinates and data recorded for plain, mountain, and alpine variables within NPAs and PAs; therefore, all of these spatial data and their associated RLL species were removed from Seriation, Kruskal–Wallis, one-way ANOSIM, and one-way NPMANOVA analyses.

Comparisons of the total species number between the NPAs and PAs was performed using the chi-squared test^56^.

*Seriation analysis* was used based on a binary data matrix that represents the presence/absence of RLL species within geomorphological and biogeographical units in Romania for both NPAs and PAs. Seriation analysis was applied to identify the chronological pattern in RLL species across geomorphological and biogeographical units. Thus, this analysis was used to reveal the (a) ordination and differences of RLL species along spatial (geomorphological and biogeographical units) and time (semicentenary periods of time) gradients, and (b) how the occurrences of RLL species are ordered along these gradients. The time periods are an important informational support over time because represent a time landmark for RLL species occurrences from spatial point of view. Furthermore, seriation analysis was used to highlight the changes in RLL species occurrences along biogeographical and geomorphological gradients throughout the time periods. The seriation analysis was performed using constrained and unconstrained algorithms^56^. Thus, in the constrained algorithm, the RLL species were chronological ordered (Table 1) whilst in the unconstrained algorithm, the RLL species were ordered along the representative period of time and its associated geomorphological and biogeographical units (Tables 2, 3). A Monte Carlo simulation was run using the constrained algorithms^56^.

The Kruskal–Wallis test was used to analyse differences within NPAs and PAs based on all of their attributes, while one-way ANOSIM was used to reveal the differences between PAs and NPAs based on all of their attributes.

*The Kruskal–Wallis* test was used to identify the differences in the occurrences of RLL species along geomorphological and biogeographical units throughout the time periods within the NPAs and PAs. A post hoc pairwise Mann–Whitney test based on *p* values was used to compare the occurrences of the RLL species along the spatial and time gradients in the NPAs and PAs^56^.

*One-way ANOSIM* was used to test the differences between the data represented by NPAs and PAs (studied groups). The significance of the test was computed by permutation of group elements with 9999 replicates. As a distance measure, the Jaccard distance index was chosen since it is appropriate for presence/absence data. A post hoc test was performed to identify differences in all pairs of studied groups represented by NPAs and PAs data^56^.

Different time periods were statistically compared; therefore, collection rates at different time periods were calculated for both NPAs and PAs. The collection rates were calculated as follows: the number of RLL species records in the biogeographical and geomorphological units relative to the unit of time, namely, 50 years^63^. The collection rates for NPAs and PAs were calculated for each period of time taken into account within this study (supplementary file: Tables S7 and Table S8). The statistical analysis of collection rates within the NPAs and PAs was performed based on a distance matrix calculated using the Gower distance index between all pairs of time periods across the biogeographical and geomorphological attributes^56^. The significance of the differential collection rates between all pairs of time periods and their geographical and geomorphological variables was tested using the Kolmogorov–Smirnov test^56^.

One-way NPMANOVA was used to test the differential collection rates between the NPAs and PAs, each of which was represented by a distance matrix that included the periods of time and their biogeographical and geomorphological variables. For each distance matrix, we selected the Gower distance index. Permutation was performed with 9999 replicates^56^.

Statistical analyses such as Seriation, Kruskal–Wallis, one-way ANOSIM, and one-way NPMANOVA were performed with PAST software^56^. Generalized estimating equations were performed with R software^59^.

## Supplementary Information

Supplementary Information.

## Data Availability

The datasets generated during and/or analysed during the current study are available from the corresponding author on reasonable request.
