# Author Correction: A biogeographical study of red listed lichen species at temporal and spatial scales within protected and non-protected areas

**DOI:** 10.1038/s41598-022-06164-0

**Published:** 2022-01-31

**Authors:** Ioana Vicol, Simona Mihăilescu

**Affiliations:** Department of Ecology, Taxonomy and Nature Conservation, Institute of Biology Bucharest of Romanian Academy, 296 Splaiul Independentei, P.O. Box 56–53, 060031 Bucharest, Romania

Correction to: *Scientific Reports* 10.1038/s41598-022-04872-1, published online 18 January 2022

The original version of this Article contained errors in the spelling of the authors Ioana Vicol and Simona Mihăilescu, which were incorrectly given as Vicol Ioana and Mihăilescu Simona.

The Author contribution statement now reads:

“I.V. contributed to conceptualization, worked in the field, collected the data, contributed with data and analysis tools, conceived and designed the statistical analysis, performed the statistical analysis, interpreted the data for the work, wrote the original draft, and critically revised the manuscript. S.M. supervision, project administration, funding acquisition, revised the original draft and revised the manuscript.”

The original Article and its accompanying Supplementary Information file have been corrected.

