# Supplementary material for: A biogeographical study of red listed lichen species at temporal and spatial scales within protected and non-protected areas
Source: Sci Rep. 2022 Jan 18;12:898. doi: 10.1038/s41598-022-04872-1 (PMC8766530; doi:10.1038/s41598-022-04872-1)
Supplement: Supplementary file 1 — Supplementary Information. [file 41598_2022_4872_MOESM1_ESM.pdf]

Supplementary Materials

A biogeographical study of red listed lichen species at temporal and spatial scales within protected and non-protected areas

Ioana Vicol[1*], Simona Mihăilescu[1]

[1]Institute of Biology Bucharest of Romanian Academy, Department of Ecology, Taxonomy and Nature Conservation, 296 Splaiul Independentei, 060031 Bucharest, P.O. Box 56-53, Romania, tel.+40213153074, fax: +40213143508, [1]e-mail: ioana21vicol@gmail.com, [1]e-mail: simona.mihailescu@gmail.com *Corresponding author

The list of supplemetary information materials

Table S1. Bibliographical and herbarium sources with regard to red listed lichen species within protected areas

Table S2. Bibliographical and herbarium sources with regard to red listed lichen species within non-protected areas

Table S3. The distribution and the number of red listed lichen species according to their occurrences in geomorphological units and biogeographical regions

Table S4. The occurrences of the RLL species within the geomorphological units and biogeographical regions over the time periods within the NPAs (the presence of the RLL is given in bold)

Table S5. The occurrences of the RLL species within the geomorphological units and the biogeographical regions over the time periods within the PAs (the presence of the RLL is given in bold)

Table S6. Red listed lichen species designated for Romania according to the references

Table S7. Collection rates calculated for the protected areas based on the biogeographical regions and the geomorphology of the studied areas during the time periods

Table S8. Collection rates calculated for the non-protected areas based on the biogeographical regions and geomorphology of the study areas during the periods of time

Table S1. Bibliographical and herbarium sources as regard red listed lichen species within protected areas in Romania

Species	References and herbarium (BUCM L) sources
Bryoria lanestris (Ach.) Brodo & D. Hawksw	[1, 2, 3, 4]
Cetraria islandica subsp. *islandica* (L.) Ach.	[2, 3, 4, 5, 6, 7, 8, 9, 10, 11, 12, 13, 14, 15, 16, 17, 18, 19, 20, 21, 22, 23, 24, 25, 26, 27, 28, 29, 30, 31, 32, 33, 34]; BUCM L09329; BUCM L0111; BUCM L0154; BUCM L0469; BUCM L0470; BUCM L0472; BUCM L0474; BUCM L0475; BUCM L0476; BUCM L0477; BUCM L0479; BUCM L0482; BUCM L0483; BUCM L0485; BUCM L0486; BUCM L0487; BUCM L0488; BUCM L0490; BUCM L0493; BUCM L0498; BUCM L3155; BUCM L3201; BUCM L3204; BUCM L3210; BUCM L3215; BUCM L3219; BUCM L3231; BUCM L3234; BUCM L3247; BUCM L3271; BUCM L3272; BUCM L3273; BUCM L3280; BUCM L3297; BUCM L3301; BUCM L3305; BUCM L3308; BUCM L3315
Cetraria sepincola (Hoffm.) Ach.	[2, 3, 6, 23, 25, 27, 28, 30, 31, 32, 33, 34, 35]; BUCM L2429; BUCM L2449
Cladonia incrassata Flörke	[2, 3, 4]
Cladonia macrophylla (Schaer.) Stenh.	[2, 3, 4, 36]
Cladonia sulphurina (Michx.) Fr.	[2, 3, 4, 27, 28, 30, 31, 34]
Dolichousnea longissima (Ach.) Articus	[2, 5, 7, 12, 18, 20, 21, 24, 28, 37, 38, 39]; BUCM L09792; BUCM L09794; BUCM L09795; BUCM L09798; BUCM L09800; BUCM L09801; BUCM L09802; BUCM L09804; BUCM L09806
Hypotrachyna sinuosa (Sm.) Hale	[2, 33, 35, 38, 39, 40, 41, 42]; BUCM L1363; BUCM L1389; BUCM L1616; BUCM L1810; BUCM L1930; BUCM L2081
Lathagrium dichotomum (With.) Otálora, P.M. Jørg. &	[2]

Wedin	
Lobaria pulmonaria (L.) Hoffm.	[2, 3, 4, 5, 6, 7, 9, 12, 16, 18, 19, 21, 23, 24, 29, 32, 33, 35, 43, 44, 45, 46, 47, 48, 49, 50, 51]; BUCM L09719; BUCM L09720; BUCM L09721; BUCM L09722; BUCM L09723; BUCM L09724; BUCM L09726; BUCM L09728; BUCM L09729; BUCM L09730; BUCM L09732; BUCM L09733; BUCM L09734; BUCM L0022; BUCM L0110; BUCM L0017; BUCM L0018; BUCM L0189; BUCM L1277; BUCM L1279; BUCM L1282; BUCM L1283
Melanelixia subaurifera (Nyl.) O. Blanco, A. Crespo, Divakar, Essl., D. Hawksw. & Lumbsch	[2, 3, 4, 6, 23, 29, 32, 49, 50, 52, 53, 54]
Nephromopsis chlorophylla (Willd.) Divakar, A. Crespo & Lumbsch	[2, 3, 4, 19, 23, 29, 38]
Peltigera lepidophora (Vain.) Bitter	[3, 4, 31, 32]
Ramalina obtusata (Arnold) Bitter	[2, 23, 52, 55, 56, 57]; BUCM L1209; BUCM L1210
Stereocaulon alpinum Laurer	[2, 3, 4, 6, 9, 14, 22, 24, 27, 58, 59, 60]; BUCM L0176; BUCM L2642; BUCM L2646; BUCM L2648; BUCM L3259
Tuckneraria laureri (Kremp.) Randlane & A. Thell	[2, 23, 31, 35, 61]
Usnea fulvoreagens (Räsänen) Räsänen	[2, 3, 24, 62]; BUCM L09805
Usnocetraria oakesiana (Tuck.) M.J. Lai & J.C. Wei	[2, 50, 62]

Table S2. Bibliographical and herbarium sources as regard red listed lichen species within non-protected areas in Romania

Species	Sources
Bryoria lanestris (Ach.) Brodo & D. Hawksw	[2]
Cetraria islandica subsp. *islandica* (L.) Ach.	[2, 9, 11, 34, 63, 64, 65, 66]; BUCM L0490; BUCM L0494
Cetraria sepincola (Hoffm.) Ach.	[2, 32]
Cladonia incrassata Flörke	[2]
Cladonia macrophylla (Schaer.) Stenh.	[2]
Cladonia sulphurina (Michx.) Fr.	[25, 34]
Dolichousnea longissima (Ach.) Articus	[2, 9, 64]; BUCM L09796; BUCM L09793; BUCM L09799; BUCM L09803; BUCM L09806
Lobaria pulmonaria (L.) Hoffm.	[2, 16, 35, 64, 65, 67, 68, 69, 70, 71, 72, 73]; BUCM L09725; BUCM L09727; BUCM L09746; BUCM L1281
Melanelixia subaurifera (Nyl.) O. Blanco, A. Crespo, Divakar, Essl., D. Hawksw. & Lumbsch	[2]
Nephromopsis chlorophylla (Willd.) Divakar, A. Crespo & Lumbsch	[2]; BUCM L09782; BUCM L09783; BUCM L09784; BUCM L0013
Peltigera lepidophora (Vain.) Bitter	[2]
Ramalina obtusata	[2, 55]

(Arnold) Bitter	
Stereocaulon alpinum Laurer	[58]
Usnea fulvoreagens (Räsänen) Räsänen	[2, 62]
Usnocetraria oakesiana (Tuck.) M.J. Lai & J.C. Wei	[2, 11]

Table S3. The distribution and the number of red listed lichen species according to their occurrences in geomorphological units and biogeographical regions within protected and non-protected areas

Protected areas											
Mountain	Hill	Plain	STE	CON	PAN	PON	ALP	1	2	3	4
18	7	4	3	9	1	1	17	7	9	17	17
Non-protected areas											
13	8	2	1	9	2	0	13	1	7	14	3

Legend: **1**-1850-1900; **2**-1901-1950; **3**-1951-2000; **4**-2001-2020; 1, 2, 3, 4, represent periods of time

Table S4. The occurrence of the RLL species within geomorphological units and biogeographical regions along time periods within NPAs (the RLL presence is given in bold)

Periods of time	Geomorpological attributes			Biogeographical regions				
	Plain	Hill	Mountain	ALP	CON	PAN	PON	STE
	Bryoria lanestris (Ach.) Brodo & D. Hawksw.							
1850-1900	0	0	0	0	0	0	0	0
1901-1950	0	0	0	0	0	0	0	0
1951-2000	0	0	**1**	**1**	0	0	0	0
2001-2020	0	0	0	0	0	0	0	0
	Cetraria islandica subsp. *islandica* (L.) Ach.							
1850-1900	0	0	0	0	0	0	0	0
1901-1950	0	0	**1**	**1**	0	0	0	0
1951-2000	0	**1**	**1**	**1**	**1**	0	0	0
2001-2020	0	0	0	0	0	0	0	0
	Cetraria sepincola (Hoffm.) Ach							
1850-1900	0	0	0	0	0	0	0	0
1901-1950	0	0	0	0	0	0	0	0
1951-2000	0	0	**1**	**1**	0	0	0	0
2001-2020	0	0	**1**	**1**	0	0	0	0
	Cladonia incrassata Flörke							
1850-1900	0	0	0	0	0	0	0	0
1901-1950	0	0	0	0	0	0	0	0
1951-2000	0	0	**1**	**1**	0	0	0	0
2001-2020	0	0	0	0	0	0	0	0
	Cladonia macrophylla (Schaer.) Stenh.							
1850-1900	0	0	0	0	0	0	0	0
1901-1950	0	0	0	0	0	0	0	0
1951-2000	0	0	**1**	**1**	0	0	0	0
2001-2020	0	0	0	0	0	0	0	0
	Cladonia sulphurina (Michx.) Fr.							
1850-1900	0	0	0	0	0	0	0	0
1901-1950	0	0	0	0	0	0	0	0

1951-2000	0	0	**1**	**1**	0	0	0	0
2001-2020	0	0	0	0	0	0	0	0
	Dolichousnea longissima (Ach.) Articus							
1850-1900	0	0	0	0	0	0	0	0
1901-1950	0	0	**1**	**1**	0	0	0	0
1951-2000	0	**1**	**1**	**1**	**1**	0	0	0
2001-2020	0	0	0	0	0	0	0	0
	Lobaria pulmonaria (L.) Hoffm.							
1850-1900	**1**	0	0	0	0	**1**	0	0
1901-1950	0	0	**1**	**1**	0	0	0	0
1951-2000	**1**	**1**	**1**	**1**	**1**	**1**	0	0
2001-2020	0	0	0	0	0	0	0	0
	Melanelixia subaurifera (Nyl.) O. Blanco, A. Crespo, Divakar, Essl., D. Hawksw. & Lumbsch							
1850-1900	0	0	0	0	0	0	0	0
1901-1950	0	0	0	0	0	0	0	0
1951-2000	0	**1**	**1**	**1**	**1**	0	0	0
2001-2020	0	0	0	0	0	0	0	0
	Nephromopsis chlorophylla (Willd.) Divakar, A. Crespo & Lumbsch							
1850-1900	0	0	0	0	0	0	0	0
1901-1950	0	0	**1**	0	**1**	0	0	0
1951-2000	0	0	**1**	**1**	0	0	0	0
2001-2020	0	0	0	0	0	0	0	0
	Peltigera lepidophora (Vain.) Bitter							
1850-1900	0	0	0	0	0	0	0	0
1901-1950	0	0	0	0	0	0	0	0
1951-2000	0	**1**	**1**	**1**	**1**	0	0	0
2001-2020	0	0	0	0	0	0	0	0
	Ramalina obtusata (Arnold) Bitter							
1850-1900	0	0	0	0	0	0	0	0
1901-1950	0	0	0	0	0	0	0	0
1951-2000	0	**1**	**1**	**1**	**1**	0	0	**1**
2001-2020	0	0	0	0	0	0	0	0

	Stereocaulon alpinum Laurer							
1850-1900	0	0	0	0	0	0	0	0
1901-1950	0	**1**	0	0	**1**	0	0	0
1951-2000	0	0	0	0	0	0	0	0
2001-2020	0	0	0	0	0	0	0	0
	Usnea fulvoreagens (Räsänen) Räsänen							
1850-1900	0	0	0	0	0	0	0	0
1901-1950	0	**1**	0	0	**1**	0	0	0
1951-2000	**1**	**1**	0	0	**1**	**1**	0	0
2001-2020	0	0	0	0	0	0	0	0
	Usnocetraria oakesiana (Tuck.) M.J. Lai & J.C. Wei							
1850-1900	0	0	0	0	0	0	0	0
1901-1950	0	0	**1**	**1**	0	0	0	0
1951-2000	0	0	**1**	**1**	0	0	0	0
2001-2020	0	0	0	0	0	0	0	0

Table S5. The occurrence of the RLL species within geomorphological units and biogeographical regions along time periods within PAs (the RLL presence is given in bold)

Periods of time	Geomorpological attributes			Biogeographical regions				
	Plain	Hill	Mountain	ALP	CON	PAN	PON	STE
	Bryoria lanestris (Ach.) Brodo & D. Hawksw.							
1850-1900	0	0	**1**	**1**	0	0	0	0
1901-1950	0	0	0	0	0	0	0	0
1951-2000	0	**1**	0	0	**1**	0	0	0
2001-2020	0	0	**1**	**1**	0	0	0	0
	Cetraria islandica subsp. *islandica* (L.) Ach.							
1850-1900	0	0	**1**	**1**	0	0	0	0
1901-1950	0	0	**1**	**1**	0	0	0	0
1951-2000	0	0	**1**	**1**	**1**	0	0	0
2001-2020	0	0	**1**	**1**	0	0	0	0
	Cetraria sepincola (Hoffm.) Ach							
1850-1900	0	0	**1**	**1**	0	0	0	0
1901-1950	0	0	**1**	**1**	0	0	0	0
1951-2000	0	0	**1**	**1**	0	0	0	0
2001-2020	0	0	**1**	**1**	0	0	0	0
	Cladonia incrassata Flörke							
1850-1900	0	0	0	0	0	0	0	0
1901-1950	0	0	0	0	0	0	0	0
1951-2000	0	**1**	**1**	**1**	**1**	0	0	0
2001-2020	0	0	**1**	**1**	0	0	0	0
	Cladonia macrophylla (Schaer.) Stenh.							
1850-1900	0	0	0	0	0	0	0	0
1901-1950	0	0	0	0	0	0	0	0
1951-2000	0	**1**	**1**	**1**	**1**	0	0	0
2001-2020	0	0	**1**	**1**	0	0	0	0
	Cladonia sulphurina (Michx.) Fr.							
1850-1900	0	0	0	0	0	0	0	0
1901-1950	0	0	0	0	0	0	0	0

1951-2000	0	0	**1**	**1**	0	0	0	0
2001-2020	0	0	**1**	**1**	0	0	0	0
	Dolichousnea longissima (Ach.) Articus							
1850-1900	0	0	**1**	**1**	0	0	0	0
1901-1950	0	0	**1**	**1**	0	0	0	0
1951-2000	0	**1**	**1**	**1**	**1**	0	0	0
2001-2020	0	0	**1**	**1**	0	0	0	0
	Hypotrachyna sinuosa (Sm.) Hale							
1850-1900	0	0	**1**	0	**1**	0	0	0
1901-1950	0	0	0	0	0	0	0	0
1951-2000	0	0	**1**	**1**	**1**	0	0	0
2001-2020	**1**	**1**	**1**	0	**1**	0	0	**1**
	Lathagrium dichotomum (With.) Otálora, P.M. Jørg. & Wedin							
1850-1900	0	0	0	0	0	0	0	0
1901-1950	0	0	0	0	0	0	0	0
1951-2000	0	0	**1**	0	**1**	0	0	0
2001-2020	0	0	0	0	0	0	0	0
	Lobaria pulmonaria (L.) Hoffm.							
1850-1900	0	0	**1**	**1**	**1**	0	0	0
1901-1950	0	0	**1**	**1**	0	0	0	0
1951-2000	**1**	**1**	**1**	**1**	**1**	**1**	0	0
2001-2020	0	0	**1**	**1**	0	0	0	0
	Melanelixia subaurifera (Nyl.) O. Blanco, A. Crespo, Divakar, Essl., D. Hawksw. & Lumbsch							
1850-1900	0	0	0	0	0	0	0	0
1901-1950	0	0	**1**	**1**	0	0	0	0
1951-2000	0	0	**1**	**1**	0	0	0	0
2001-2020	**1**	0	**1**	**1**	0	0	0	**1**
	Nephromopsis chlorophylla (Willd.) Divakar, A. Crespo & Lumbsch							
1850-1900	0	0	0	0	0	0	0	0
1901-1950	0	0	0	0	0	0	0	0
1951-2000	0	0	**1**	**1**	0	0	0	0
2001-2020	0	0	**1**	**1**	0	0	0	0

	Peltigera lepidophora (Vain.) Bitter							
1850-1900	0	0	0	0	0	0	0	0
1901-1950	0	0	0	0	0	0	0	0
1951-2000	0	0	0	0	0	0	0	0
2001-2020	0	0	**1**	**1**	0	0	0	0
	Ramalina obtusata (Arnold) Bitter							
1850-1900	0	0	0	0	0	0	0	0
1901-1950	**1**	0	0	0	**1**	0	0	0
1951-2000	**1**	**1**	**1**	**1**	**1**	0	**1**	**1**
2001-2020	0	0	**1**	**1**	0	0	0	**1**
	Stereocaulon alpinum Laurer							
1850-1900	0	0	0	0	0	0	0	0
1901-1950	0	0	**1**	**1**	0	0	0	0
1951-2000	0	0	**1**	**1**	0	0	0	0
2001-2020	0	0	**1**	**1**	0	0	0	0
	Tuckneraria laureri (Kremp.) Randlane & A. Thell							
1850-1900	0	0	**1**	**1**	0	0	0	0
1901-1950	0	0	0	0	0	0	0	0
1951-2000	0	0	**1**	**1**	0	0	0	0
2001-2020	0	0	**1**	**1**	0	0	0	0
	Usnea fulvoreagens (Räsänen) Räsänen							
1850-1900	0	0	0	0	0	0	0	0
1901-1950	0	0	**1**	**1**	0	0	0	0
1951-2000	0	0	**1**	**1**	0	0	0	0
2001-2020	0	0	**1**	**1**	0	0	0	0
	Usnocetraria oakesiana (Tuck.) M.J. Lai & J.C. Wei							
1850-1900	0	0	0	0	0	0	0	0
1901-1950	0	0	**1**	**1**	0	0	0	0
1951-2000	0	0	**1**	**1**	0	0	0	0
2001-2020	0	0	**1**	**1**	0	0	0	0

Table S6. Red listed lichen species designated for Romania according to references

Nr. crt.	Species	Reference sources
01	*Bryoria lanestris* (Ach.) Brodo & D. Hawksw.	[3]
02	*Cetraria islandica* subsp. *islandica* (L.) Ach.	[3]
03	*Cetraria sepincola* (Hoffm.) Ach.	[3]
04	*Cladonia incrassata* Flörke	[3]
05	*Cladonia macrophylla* (Schaer.) Stenh.	[3]
06	*Cladonia sulphurina* (Michx.) Fr.	[3]
07	*Dolichousnea longissima* (Ach.) Articus	[74]
08	*Hypotrachyna sinuosa* (Sm.) Hale	[74]
09	*Lathagrium dichotomum* (With.) Otálora, P.M. Jørg. & Wedin	[74]
10	*Lobaria pulmonaria* (L.) Hoffm.	[3]
11	*Melanelixia subaurifera* (Nyl.) O. Blanco, A. Crespo, Divakar, Essl., D. Hawksw. & Lumbsch	[3]
12	*Nephromopsis chlorophylla* (Willd.) Divakar, A. Crespo & Lumbsch	[3]
13	*Peltigera lepidophora* (Vain.) Bitter	[3]
14	*Ramalina obtusata* (Arnold) Bitter	[74]
15	*Stereocaulon alpinum* Laurer	[3]
16	*Tuckneraria laureri* (Kremp.) Randlane & A. Thell	[74]
17	*Usnea fulvoreagens* (Räsänen) Räsänen	[3]
18	*Usnocetraria oakesiana* (Tuck.) M.J. Lai & J.C. Wei	[74]

Table S7. Collection rates calculated for non-protected areas based on biogeographical regions and geomorphology of the studies areas during periods of time

Periods of time	Geomorpological attributes			Biogeographical regions				
	Plain	Hill	Mountain	ALP	CON	PAN	PON	STE
1850-1900	0.02	0	0	0	0	0.02	0	0
1901-1950	0	0.06	0.32	0.3	0.08	0	0	0
1951-2000	0.1	0.74	1.72	1.58	0.9	0.04	0	0.02
2001-2020	0	0	0.02	0.02	0	0	0	0

Table S8. Collection rates calculated for protected areas based on biogeographical regions and geomorphology of the studied areas during time periods

Periods of time	Geomorpological attributes			Biogeographical regions				
	Plain	Hill	Mountain	ALP	CON	PAN	PON	STE
1850-1900	0	0	0.22	0.16	0.06	0	0	0
1901-1950	0.04	0	1.24	1.24	0.04	0	0	0
1951-2000	0.08	0.22	4.96	4.72	0.48	0.02	0.02	0.02
2001-2020	0.06	0.06	3.36	3.28	0.1	0	0	0.1

References

1. Hazslinszky, F. Die alpine Florae der Alpe Pietroz bei Borșa. *Bot. Ztg*. **26**, 129-153 (1868).

2. Moruzi, C., Petria, E. & Mantu, E. Catalogul Lichenilor din România. *Acta Horti Bot. Bucurest.*: 1- 389 (1967).

3. Ardelean, I. V., Keller, C. & Scheidegger, C. Lichen flora of Rodnei Mountains National Park (Eastern Carpathians, Romania) including new records for the Romanian mycoflora. *Folia Cryptogam. Estonica*. **50**, 101-115. https://doi.org/10.12697/fce.2013.50.13 (2013).

4. Ardelean, I. V., Keller, C. & Scheidegger, C. Effects of management on lichen species richness, ecological traits and community structure in the Rodnei Mountains National Park (Romania). *PLoS ONE*. **10**(12), e0145808. https://doi.org/10.1371/journal.pone.0145808 (2015).

5. Barth, J. Eine botanische excursion in's Hátszegerthal, dann in die beiden Schielthäler und auf das Páreng-oder Parângul-Gebirge vom 22. bis 26. August 1882. *Verh. Mitth. Siebenbürg. Vereins Naturwiss. Hermannstadt* **33**, 1-10 (1883).

6. Zschacke, H. Zur Flechtenflora von Siebenbbürgen. *Verh. Mitth. Siebenbürg. Vereins Naturwiss. Hermannstadt* **63**(4-5), 111-166 (1913).

7. Borza, A. & Țenchea, V. Flora Stânei de Vale III. Lichenii. *Bul. Grad. Bot. Univ. Cluj*. **26**(1-2), 10-11 (1946).

8. Cretzoiu, P. & Klement, P. Stațiuni interesante de licheni din România. *Revista Ştiințifică V. Adamachi*. **21**(4), 206-207 (1935).

9. Cretzoiu, P. Lichenii din herbarul Al. Borza, colectați de J. Barth. *Bul. Grad. Bot. Univ. Cluj*. **19**, 122-125 (1939a).

10. Cretzoiu, P. Lichenii colectati de E. I. Nyárády. *Bul. Grad. Bot. Univ. Cluj*. **19**(1-2), 104-108 (1939b).

11. Cretzoiu, P. Contribuțiuni lichenologice din Herbarul Muzeului Botanic al Universității din Cluj. *Bul. Grăd. Bot. Muz. Bot. Cluj*. **20**(3-4), 97-126 (1941c).

12. Ștefureac, T.I. Cercetări sinecologice și sociologice asupra bryophytelor din Codrul Secular Slătioara (Bucovina). *Acad. Româna, Mem. Sect. Sti*. **14** (3), 1133-1329 (1941).

13. Codoreanu, V. Contribuţiuni la studiul florei lichenologice a Munţilor Călimani. *Studii şi Cerc. Şt. Acad. R.P.R. Filiala Cluj*. **1–2**, 170–177 (1952).

14. Codoreanu, V. Noi contribuții la flora lichenologică a Munților Făgăraș. *Bul. ştiinţ. - Acad. Repub. Pop. Rom., Secţ. biol. ştiinţe agric., Ser. bot*. **9**(4), 339-350 (1957).

15. Codoreanu, V. & Ciurchea, M. Contribuții la cunoașterea florei lichenologice de pe șisturi cristaline. *Stud. cercet. biol., Ser. Bot.* **17**(2), 145-151 (1965).
16. Burlacu, L. Contribuții la cunoașterea florei lichenologice din Cheile Bicazului. *Comun. Bot.* **9**, 207-215 (1969a).
17. Burlacu, L. Contribuții la cunoașterea florei lichenologice din Moldova. *Comun. Bot.* **8**, 79-88 (1969b).
18. Burlacu, L. & Diaconescu, F. Contributii la cunoasterea florei lichenologice din Masivul Leaota. *Analele Şti. Univ. "Al. I. Cuza" Iaşi, Ser. Nouă 2.a.* **15**(1), 203-209 (1969).
19. Bartók, K. & Codoreanu, V. Contribuții la cunoasterea florei și vegetației lichenologice din Munții Vlădeasa (rama vestică). *Contrib. Bot.*: 37-45 (1979).
20. Bartók, K. Recherches lichénologiques dans quelques forêts d' picéa de la Transylvanie (Roumanie). *Contrib. Bot.*: 111-116 (1989).
21. Bartók, K. Structura si biomasa comunitatilor de licheni (Lichenophyta) in *Parcul National Retezat, Studii ecologice*: 136-147 (Editura Vest Side Computers Brasov, 1993).
22. Ciurchea, M. & Crișan, F. Vegetația lichenologică saxicolă din Rezervația Științifică a Parcului Național Retezat in *Parcul Național Retezat, Studii ecologice*: 58-77 (Editura West Side Computers Brasov, 1993).
23. Manoliu, A., Negrean, G., Zanoschi, V., Monah, F. & Coroi, M. *Plante inferioare din Masivul Ceahlău. Alge, ciuperci, licheni, mușchi* (Cermi Publishing House Iași, 1998).
24. Zamfir, M., Mărgăritescu, S. & Moberg, R. Contribution to the lichen flora of Romania 1. The lichen flora of Bucegi Mts. (Muntenia). *Herzogia* **13**: 189-197 (1998).
25. Stoie, A. Studiul floristic asupra lichenilor foliacei şi fruticuloşi din rezervaţia Bistricioru (M. Călimani). *Stud. Cercet., Biol., Bistriţa* **5**, 97–103 (1999).
26. Ciurchea, M. *Determinatorul lichenilor din România* (Editura Bit Iași, 2004).
27. Bartók, K. & Lőkös, L. Contributions to the lichen flora and lichen ecology in the Parâng and Lotru mountains (Southern Carpathians). *Contrib. Bot.* **39**, 41-49 (2004).
28. Popa, L. Contributions to the study of the lichen flora from Bistrița Mountains. *Bul. Grăd. Bot. Iași* **36**, 119-127 (2006).
29. Çobanoğlu, G. *et al*. Epiphytic and terricolous lichens diversity in Cozia National Park (Romania). *Oltenia. Studii comunicări. Științele naturii* **25**, 17-22 (2009).
30. Crișan, F. & Ardelean, I. The analysis of ecological behavior of the macrolichens from Rosia Montana (Romania). *Studia Univ. Vasile Goldis Arad, Ser. Stiintele Vietii* **20**(4), 53-56 (2010).

31. Vondrák, J. & Liška, J. Lichens and lichenicolous fungi from the Retezat Mts and overlooked records for the checklist of Romanian lichens. *Herzogia* **26**(2), 293-305. https://doi.org/10.13158/heia.26.2.2013.293 (2013).
32. Malíček, J., Bouda, F., Liška, J., Palice, Z. & Peksa, O. Contribution to the lichen biota of the Romanian Carpathians. *Herzogia* **28** (2), 713-735. https://doi.org/10.13158/heia.28.2.2015.713 (2015).
33. Vicol, I. Red listed lichen species within old growth and young growth forests from Romania. *Rom. J. Biol. - Plant Biol.* **62**(2), 67-76 (2017).
34. Lőkös, L., Crișan, F., Hur, J. S., Varga, N., Farkas, E. Enumeration of the lichen forming and lichenicolous funghi of the Călimani Mountains (Eastern Carpathians, Romania). *Stud. Bot. Hung.* **49**(1), 5-40. https://doi.org/10.17110/StudBot.2018.49.1.5 (2018).
35. Hazslinszky, F. *A Magyar birodalom Zusmóflórája* (Kiadja A.K.M. Természettudományi társulat, 1884).
36. Mantu, E. & Sava, G. Lichenii din Bazinul Uzului, regiunea Munților Nemirei și Ciucului (nota 1). *Studii și Comunicări, Muzeul Bacău*: 246-258 (1969).
37. Servit, M., Hillman, J., Erichsen, C.F.F., Cretzoiu, P. Zur Kenntnis der Flechtenflora von Rumänien. *Feddes Repert. Spec. Nov. Regni Veg.* **36**, 289-299 (1934).
38. Bartók, K. Recherches lichénologiques dans quelques forêts de mélanges (hétres et résineuses) de la Transylvanie (Roumanie). *Contrib. Bot.*: 189-196. (1988).
39. Bartók, K. Studiul florei de licheni din Sud-estul Munților Zarandului. *Studia Univ. Vasile Goldis Arad, Ser. Stiintele Vietii* **4**, 102-108 (1994).
40. Vicol, I. Preliminary study on epiphytic lichens as an indicator of environmental quality in forests from around Bucharest Municipality (Romania). *Analele Univ. din Oradea, Fasc. Biol.* **17**(1), 200–207 (2010).
41. Vicol, I. Synecological structure of the lichen synusiae within forest natural reserves from the Moldavian Plateau (Romania). *Turk. J. Bot.* **39**(1), 189–197 (2015b).
42. Vicol, I. Ecological patterns of lichen species abundance in mixed forests of Eastern Romania. *Ann. For. Res.* **59**(2), 237–248. http://doi:10.3906/bot-1312-10 (2016).
43. Brândză, D. *Prodromul florei Române sau enumerațiunea plantelor pănă astă-ḍi cunoscute în Moldova și Valahia*. Bucuresci (Tipografia Academiei Române, 1879-1883).
44. Mantu, E. Lichens associations from Ciucaș and Bucegi Mts. *Trav. Mus. Natl. Hist. Nat. "Grigore Antipa"* **7**, 475-481 (1967).
45. Moruzi, C. & Toma, N. Contribuții la cunoașterea florei lichenologice corticole, tericole și muscicole din zona Portilor de Fier (I). *Acta Horti Bot. Bucurest*: 433-466 (1970-1971).

46. Codoreanu, V. & Micle, F. Flora și vegetația lichenologică de pe Valea Sebișelului (Munții Apuseni). *Contrib*. *Bot*.: 59-73 (1976).
47. Bartók, K. & Codoreanu, V. Compoziția lichenologică a cenozelor din Masivul Vlădeasa (Bazinul Drăganului și Iadului). *Nymphaea* (Oradea) **10**, 207-216 (1983).
48. Bartók, K. Lichen biomass of dwarf pine shrubberies in the Retezat National Park. *Stud*. *Cercet*. *Biol*. *(Bucharest), Ser*. *Biol*. *Veg*. **32**(2), 91- 96 (1987).
49. Bartók, K., Guttova, A. & Lőkös, L. Contributions to the epiphytic lichen flora of Poiana Ruscă Mts, Southern Carpathians (SW Romania). *Contrib*. *Bot*. **40**, 105-110 (2005).
50. Sass-Gyarmati, A., Molnár, K., Orbán, S., Pócs, T. & Erzberger, P. The cryptogamic flora of the Zugrăști sinkhole system and its surroundings (Apuseni Mountains, Romania). *Kanitzia* **16**, 25-44 (2008-2009).
51. Vicol, I. Effect of old-growth forest attributes on lichen species abundances: a study performed within Ceahlău National Park (Romania), *Cryptogamie, Mycol*. **36**(4), 399-407. https://doi.org/10.7872/crym/v36.iss4.2015.399 (2015a).
52. Crișan, F. The ecological analysis of the corticolous macrolichens from the Pădurea Craiului Mountains (Bihor Department). *Contrib*. *Bot*. **38**(1), 19-24 (2003).
53. Toma, N. & Covaliuc, M. Taxonomic diversity of lichens found on the Caraorman grind (the Danube Delta). *Acta Horti Bot. Bucurest*. **33**, 131-142 (2006).
54. Crișan, F. Contributions to the study of the corticolous lichen vegetation of the Padurea Craiului Mountains (Bihor department, Romania). *Studia Univ. Vasile Goldis Arad, Ser. Stiintele Vietii* **20**(3), 71-76 (2010).
55. Moruzi, C. & Klohs, D. Taxoni noi și rari pentru flora lichenologică a României. *Comunicări și referate Muzeul de Ştiințele Naturii Ploiești*: 105-108 (1971).
56. Pišút, I. Interessante Flechtenfunde aus Mittel-und Südeuropa. *Fragm*. *Balcan*. **8**, 165 – 169 (1971).
57. Fodor, E. Analysis of the saxicolous lichen communities in Măcin Mountains National Park. *Acta Horti Bot*. *Bucurest*. **42**, 67-86 (2015).
58. Gyelnic, V. Beiträge zur Flechtenvegetation Ungarns II. *Folia Cryptog*. **1**(6), 577-604 (1928).
59. Cretzoiu, P. Contribuțiuni lichenologice din Herbarul Muzeului Botanic al Universității din Cluj II. *Bul*. *Grăd*. *Bot*. *Univ*. *Cluj* **20**(1-2), 1-11 (1941a).
60. Moruzi, C. & Toma, N. Contribuții la cunoașterea Cladoniaceelor din Munții Cibinului. *Acta Horti Bot*. *Bucurest*.: 325-333 (1964-1965).

61. Ciurchea, M. Zur Flechtenflora und Vegetation des Muntele Mare und der Scărișoara-Belioara (Munții Apuseni). *Stud. Univ. Babeş-Bolyai, Ser. 2, Biol.* **2**, 39-49 (1967).
62. Cretzoiu, P. Flora lichenilor folioși și fruticuloși epidendri și epixili din România. *I.C.E.F. Referate-Comunicari,* Seria II, Ministerul Agriculturii si Domenii 47, Bucuresti (1941b).
63. Gușuleac, M. Considerațiuni geobotanice asupra Pinului silvestru din Bucovina. *Bul. Fac. Sti. Cernăuți.* **4**(2), 310-375 (1930).
64. Borza, A. *Flora și vegetația Văii Sebeșului* (Editura Academiei Republicii Populare Române, Bucuresti, 1959).
65. Rotărescu-Burlacu, L. Contribuțiuni la cunoașterea florei lichenologice din Munții Tarcăului. *Comun. Bot.* **2**(2), 139-145 (1963).
66. Stoie, A. & Crişan, F. Studiul floristic asupra lichenilor foliacei şi fruticuloşi din rezervaţiile "Valea Repedea" şi "Pădurea din Şes" (Transilvania de Nord-Est). *Studii şi cercetări, Ştiinţele Naturii, Bistriţa.* **4**, 141–156 (1998).
67. Burlacu, L. Contribuții la cunoașterea florei și vegetației lichenologice a pădurilor din raionul Dorohoi (reg. Suceava). *An. Stiint. Univ. Al. I. Cuza Iasi Sect. II (A) Biol.* **13**(1), 167-172 (1967).
68. Moruzi, C. & Toma, N. Noi contributii la cunoasterea lichenilor din Munții Cibinului (II). *Acta Horti Bot. Bucurest.*: 295-303 (1967).
69. Moruzi, C. & Toma, N. Contribuții la studiul vegetației lichenologice a Munților Cibinului. *Acta Horti Bot. Bucurest.*: 165-190 (1969).
70. Ciurchea, M. Cercetări asupra florei și vegetației lichenologice arboricole din regiunea Arcalia. *Contrib. Bot.*: 133-139 (1972).
71. Ciurchea, M. & Eftimie, E. Beiträge zur Kenntnis der Flechtenflora des Casin-Beckens (Kreis Bacău). *Travaux de la station "Stejarul", Pângarati*: 129-133 (1974-1975).
72. Codoreanu, V. Lichenoflora, Flora și vegetația Munților Zarand. *Contrib. Bot.*: 68-75 (1978).
73. Bartók, K. Flora și vegetația lichenologică a pădurilor de amestec din Munții Bihorului. *Stud. Cercet. Biol. (Bucharest), Ser. Biol. Veg.* **34**(2), 101-106 (1982).
74. Sârbu, A., *et al. Arii speciale pentru protecția și conservarea plantelor în România* (Victor B Victor, Bucharest, 2007).
